# RNAdjuvant^®^, a novel, highly-potent RNA-based adjuvant, combines strong immunostimulatory capacities with a favorable safety profile

**DOI:** 10.1186/2051-1426-3-S2-P163

**Published:** 2015-11-04

**Authors:** Regina Heidenreich, Keyvan Tadjalli Mehr, Janine Noth, Sven Koch, Henoch Hong, Karl Melber, Angelika Daehling, Tilmann Roos, Johannes Lutz, Aleksandra Kowalczyk, Patrick Baumhof, Birgit Scheel, Söhnke Voss, Karl-Josef Kallen, Mariola Fotin-Mleczek, Ulrike Gnad-Vogt

**Affiliations:** 1 CureVac GmbH, Tuebingen, Germany; 2 CureVac GmbH, Tübingen, Germany; 3 CureVac GmbH Frankfurt, Frankfurt, Germany

## 

Purified recombinant proteins and peptides, which are currently under development in various anti-cancer vaccination approaches, lack sufficient immunogenicity. Therefore, potent adjuvants are needed to induce strong and persistent anti-tumor immunity. However, currently only few adjuvants are licensed, most of which primarily enhance antibody, but not T cell responses.

Here, we demonstrate that a novel, well defined, and thoroughly characterized RNA-based adjuvant mediates balanced and long-lasting humoral and cellular immune responses. Our adjuvant significantly enhances anti-tumor immunity, and even complete tumor rejection can be achieved as shown for the syngeneic TC-1 tumor model, a murine model of human HPV-induced cervical cancer.

Our adjuvant acts locally, promoting strong but transient up-regulation of anti-viral and pro-inflammatory cytokines, CXCR3-ligands and cytoplasmic RNA sensors at the injection site, avoiding any systemic cytokine release. These changes are followed by activation of different subsets of immune cells in the draining lymph nodes. In repeated dose toxicity studies carried out in mice and pigs no toxicity events were observed demonstrating an excellent preclinical safety profile. A Phase I first in man clinical trial testing different doses of RNAdjuvant^®^ alone and in combination with reduced doses of the licensed rabies vaccine Rabipur^®^ is currently ongoing. Healthy volunteers receive 2 intramuscular injections on days 0 and 21 either with RNAdjuvant^®^ alone or in combination with 1/20 or 1/10 of the licensed Rabipur^®^ dose. In both groups vaccinations were well tolerated with mild to moderate injection site reactions and flu-like symptoms as main side effects. Virus neutralizing antibody titers (VNTs) are measured on days 14 and 28 and a significant increase in median VNTs is observed after vaccination with 1/10 dose Rabipur^®^ / RNAdjuvant^®^ compared to 1/10 dose Rabipur^®^ alone. In summary, our data suggest that RNAdjuvant^®^ represents a novel, highly efficacious adjuvant candidate that can enhance cellular and humoral immune responses.

Preliminary results of a first in human trial show a favorable safety profile and enhancement of immune responses in combination with a licensed rabies vaccine indicating an antigen sparing effect.

While the field of cancer vaccination is currently obstructed by a lack of potent and safe adjuvants, RNAdjuvant^®^ has the potential to fill this gap and to improve the efficacy of many cancer vaccines.

